# Supplementary material for: Integrated Proteomics and Metabolomics Link Acne to the Action Mechanisms of Cryptotanshinone Intervention
Source: Front Pharmacol. 2021 Sep 1;12:700696. doi: 10.3389/fphar.2021.700696 (PMC8440807; doi:10.3389/fphar.2021.700696)
Supplement: Supplementary file 1 [file DataSheet1.ZIP › Supplementary table 1-10 and material 1-3/Material 3-Metlib-MSMS/NEG-Metlib-MSMS/Metlib-MSMS/M179T347_forward/0.684,Glyceraldehyde,(2M-H)-.pdf]

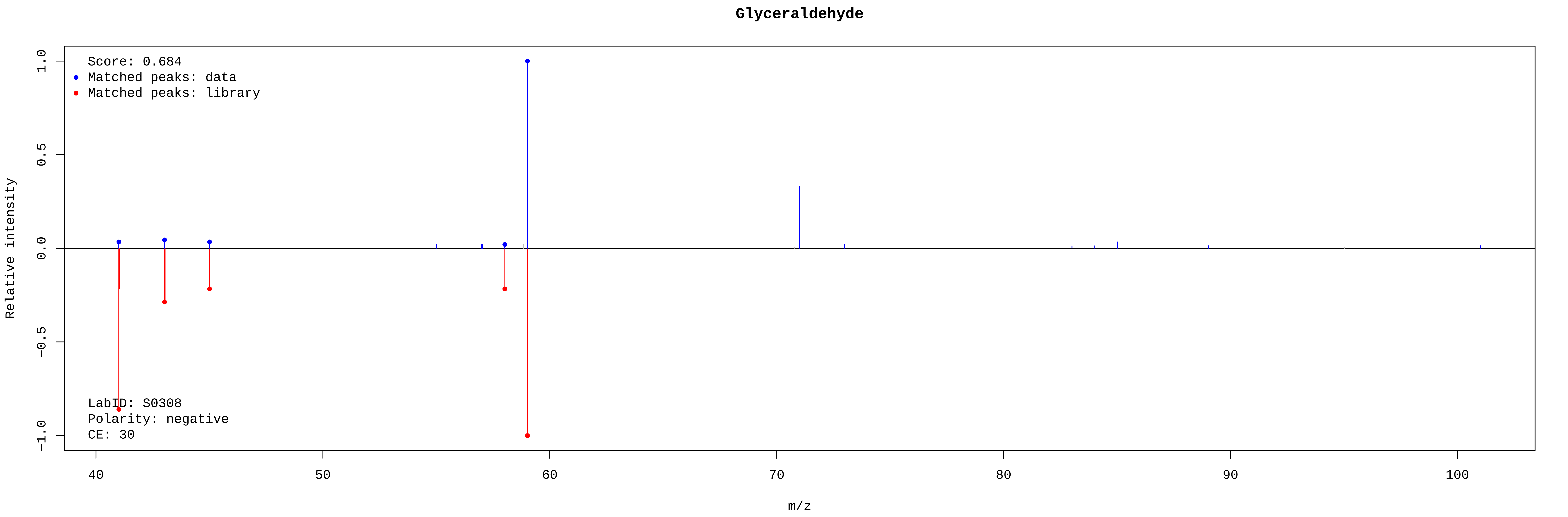
Glyceraldehyde
Score: 0.684
Matched peaks: data
Matched peaks: library
LabID: S0308
Polarity: negative
CE: 30
Relative intensity
1.0
0.5
0.0
−0.5
−1.0
40
50
60
70
80
90
100
m/z
